# Early Diagnosis of Male Breast Cancer: A Case Report and Literature Review

**DOI:** 10.7759/cureus.100241

**Published:** 2025-12-28

**Authors:** José Aderval Aragão, José Valdercides Amaral, Iapunira Catarina Sant’Anna Aragão, Felipe Matheus Sant’Anna Aragão, Francisco Prado Reis

**Affiliations:** 1 Morphology and Medicine, Federal University of Sergipe (UFS), Aracaju, BRA; 2 Mastology, IMAMA - Digital Mammography and Various Ultrasound Services, Aracaju, BRA; 3 Internal Medicine, Munir Rafful Municipal Hospital, Volta Redonda, BRA; 4 Cardiology, Hospital das Clínicas, University of São Paulo, São Paulo, BRA; 5 Medicine, Medical School of Tiradentes University, Aracaju, BRA

**Keywords:** androgen receptor, brca1, brca2, diagnosis, genetic factor, male breast cancer, multigene panel testing, prognosis, risk factors, treatment

## Abstract

Male breast cancer (MBC) is a rare neoplasm, and this rarity underscores the critical importance of awareness and appropriate screening. This study presents a case of male breast cancer (MBC) in a 64-year-old patient to enhance understanding of this rare and frequently underdiagnosed neoplasm. A 64-year-old man presented with a palpable mass in the left breast. Imaging revealed bilateral gynecomastia and a highly suspicious 4.3-cm Breast Imaging Reporting and Data System (BI-RADS) 5 nodule with nipple retraction. Biopsy confirmed histologic grade III invasive ductal carcinoma. Immunohistochemistry showed positivity for hormone receptors (Estrogen Receptor (ER) 90%, Progesterone Receptor (PR) 50%) and was negative for Human Epidermal Growth Factor Receptor 2 (HER2). Following left mastectomy, the patient experienced postoperative complications, including a contralateral hematoma and a persistent seroma. Oncotype diagnosis (DX) testing indicated a low chemotherapy benefit, leading to adjuvant therapy with tamoxifen. Pathologic tumor stage pT1c and pathologic nodal status pN0, confirmed by sentinel lymph node biopsy, indicated early-stage disease. The complexity of MBC underscores the critical importance of early diagnosis. The prompt detection of the suspicious mass, culminating in effective early-stage treatment, proved crucial to this patient’s favorable prognosis, in contrast to the common trend of delayed diagnoses and their less promising outcomes. Accordingly, strengthening public awareness and professional training is essential to optimize management and improve outcomes in men with breast cancer.

## Introduction

Male breast cancer (MBC) is a rare neoplasm, accounting for a small fraction (approximately 0.5% to 1%) of all breast cancer diagnoses worldwide [1,2]. Despite its low prevalence, the incidence of MBC has shown a progressive increase over recent decades, generating growing interest and concern within the oncology community [3-5]. Epidemiologically, men are typically diagnosed later in life, with a mean age between 60 and 70 years, and they frequently present with more advanced disease stages at diagnosis [6]. This delayed presentation contributes to a generally less favorable prognosis compared with female breast cancer (FBC) [7-9]. In male breast cancer (MBC), several parameters have been identified as prognostic factors, including race, lymph node involvement, tumor size, androgen receptor status, histologic grade, and age at diagnosis [10-12]. The poorer prognosis of MBC is largely related to advanced stage at presentation, driven by low awareness and higher rates of lymph node metastasis. Although lymph node metastasis may be present, limited involvement (≤3 lymph nodes) does not preclude a favorable prognosis; in contrast, involvement of more than three lymph nodes is associated with an increased risk of systemic metastasis and poorer clinical outcomes [13].

The etiology of MBC is complex and multifactorial, involving a range of genetic and environmental risk factors. Among genetic predispositions, pathogenic variants in genes, such as BRCA2 (the most prevalent), BRCA1, PALB2, CHEK2, and ATM, in addition to a positive family history of breast cancer, are noteworthy [14-19]. Hormonal imbalances, notably hyperestrogenism, frequently associated with Klinefelter syndrome, obesity, liver disease, and exogenous estrogen therapy and radiation exposure, are significant etiological factors in MBC [15,20-22]. Furthermore, advanced age has been identified as a risk factor for MBC. Conditions that induce endocrine imbalance, resulting in an elevated estrogen-to-androgen ratio, are also recognized contributors to the risk of MBC. Examples include obesity (BMI > 30, associated with a nearly doubled risk), Klinefelter syndrome (3% of MBC cases), the use of certain medications, and exogenous hormonal therapy (e.g., in gender transition). Testicular dysfunctions, such as mumps orchitis, cryptorchidism, and testicular neoplasms, are also associated with an increased risk of MBC [3].

Clinically, male breast cancer (MBC) most commonly presents as a palpable, usually painless breast mass, frequently located in the retroareolar region, and it may be accompanied by skin retraction or nipple discharge [16,23]. Histopathologically, the predominant histologic type is invasive ductal carcinoma (IDC), characterized by high positivity for hormone receptors, specifically the estrogen receptor (ER) and progesterone receptor (PR) (present in more than 90% of cases). Conversely, HER2 (human epidermal growth factor receptor 2) positivity and the incidence of triple-negative breast cancer (TNBC) are considerably low [2,3,24,25]. TNBC is an aggressive and heterogeneous molecular subtype of breast cancer, characterized by the absence of estrogen receptor (ER), progesterone receptor (PR), and HER2 expression. Its intrinsic aggressiveness, unfavorable prognosis, and lack of conventional molecular targets render its therapeutic management a significant challenge, with the majority of cases classified as the basal-like subtype [26].

Although this tumor biology shares similarities with the luminal subtype of postmenopausal female breast cancer, molecular investigations have revealed particular features that distinguish MBC from female breast cancer [27]. The MBC is predominantly hormonally sensitive, with the majority of cases exhibiting estrogen receptor positivity. The most common histology, similar to female breast cancer, is invasive ductal carcinoma; however, papillary carcinomas are comparatively more frequent, and lobular carcinomas are rarer in men. Conclusive evaluation of histological grade and HER2 status is challenged by the limitation of retrospective data and the variability in findings for HER2 overexpression and tumor grade. Research indicates additional biological particularities in MBC, including a higher frequency of p53-negativity (Proteína Tumoral 53), p21-positivity (Cyclin-Dependent Kinase Inhibitor 1A), and aneuploidy. Potential relevance of kinase inhibitor proteins, increased activity of androgenic pathways (evidenced by high androgen receptor (AR) positivity), and involvement of the prolactin receptor in carcinogenesis are also observed [28-31].

The therapeutic management of MBC, due to its rarity and the limited availability of randomized clinical trials specifically targeting male patients, is largely guided by extrapolation from protocols established for female breast cancer [32-34]. Treatment strategies primarily include surgical intervention (with mastectomy being the most common technique), adjuvant chemotherapy for high-risk cases, and endocrine therapy (predominantly tamoxifen) for most hormone-dependent tumors [15,35,36]. Oncogenetic counseling plays a crucial role given the high prevalence of hereditary genetic mutations identified in this population [37]. However, adherence to long-term endocrine therapies, such as tamoxifen, constitutes a significant challenge, often due to associated adverse effects [38,39].

This complex clinical scenario highlights the imperative need for greater awareness among the general population and healthcare professionals, as well as ongoing investment in research dedicated to MBC. Such efforts are crucial to optimize early diagnosis, refine therapeutic strategies, and consequently improve the prognosis of male patients affected by this disease. In this context, the present work aimed to report a case of male breast cancer in a 64-year-old patient, seeking to contribute to the understanding and dissemination of knowledge about this rare condition.

## Case presentation

**Figure 1:**
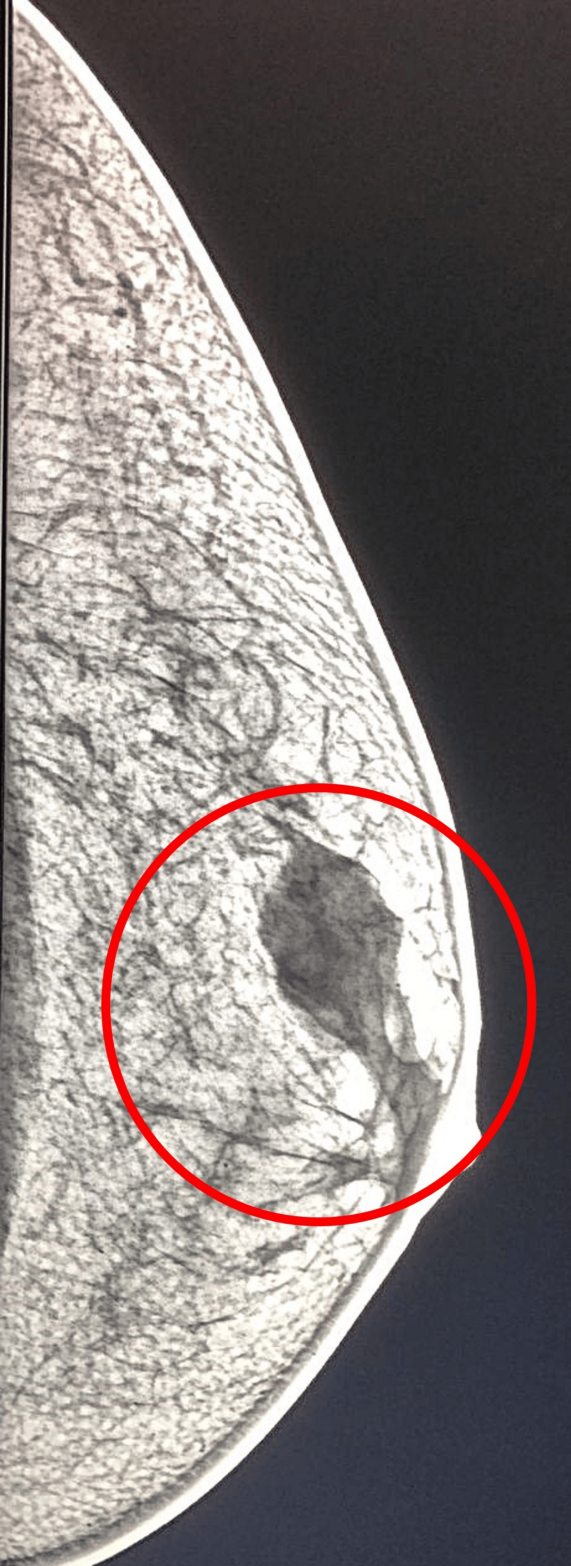
Mammography showing a nodule in the left breast.

A 64-year-old asymptomatic male patient presented with a mass in the left breast. Following palpation, breast ultrasonography was performed and, subsequently, diagnostic mammography. The examinations revealed bilateral gynecomastia and a 4.3cm nodule in the upper outer quadrant (UOQ) of the left breast, of dense and irregular consistency, extending to the nipple region with retraction of the nipple-areolar complex. This finding was classified as Category 5 (highly suspicious) according to the Breast Imaging Reporting and Data System (BI-RADS) (Figure 1) [40].

After mammography, a fine-needle aspiration (FNA) was performed, and cytology indicated atypical cells. The following day, a core needle biopsy (CNB) was conducted, yielding four filiform fragments of whitish, elastic tissue, the largest measuring 1.1 × 0.2 × 0.1 cm. Histopathological examination revealed invasive carcinoma of no special type (ductal), histologic grade III and nuclear grade 3, with no evidence of lymphovascular invasion. 

Computed tomography (CT) of the chest and abdomen identified a lobulated nodule measuring 2.4 × 1.9 cm on the axial plane in the left breast with heterogeneous enhancement and of indeterminate nature. The lesion exhibited a cleavage plane at a distance of 1.2 cm from the pectoral musculature. Abdominal CT showed hypervascular hepatic nodular outlines, interpreted as benign hepatocellular lesions.

Bone scintigraphy with single-photon emission computed tomography-computed tomography (SPECT-CT) demonstrated a focus of mild radiotracer uptake at the right L4-L5 and left L5-S1 facet joints, consistent with degenerative/inflammatory osteoarticular changes. The remainder of the skeletal framework showed no areas of abnormal uptake for the patient’s age, and there were no findings suspicious for active osteoblastic neoplastic/secondary lesions. HER2 fluorescence in situ hybridization (FISH) confirmed that the invasive carcinoma was negative for HER2 gene amplification, with a HER2/CEP17 ratio < 2 (Group 5), indicating a less aggressive profile than HER2-positive disease. Fluorodeoxyglucose positron emission tomography-computed tomography (FDG PET-CT) demonstrated increased radiotracer uptake, consistent with elevated glycolytic metabolism, in an intensely hypermetabolic solid nodule located in the upper outer quadrant of the left breast near the retroareolar region, measuring 2.8 × 2.0 cm, with a standardized uptake value (SUV) of 8.3. No additional relevant foci were identified beyond the expected physiological biodistribution for the patient’s age. Next-generation sequencing (NGS) for hereditary breast cancer panel and tumor markers was performed for a hereditary breast cancer NGS panel, along with tumor markers (CA 19-9, CA 15-3, and CEA). Myocardial perfusion scintigraphy demonstrated a stress-induced ischemic area in the anterior wall (apical and mid segments) on SPECT-CT with CT-based attenuation correction, involving approximately 7% of the left ventricular myocardium. Lymphoscintigraphy identified more than five radiotracer-avid foci in the left axillary region during sentinel lymph node mapping.

**Figure 2:**
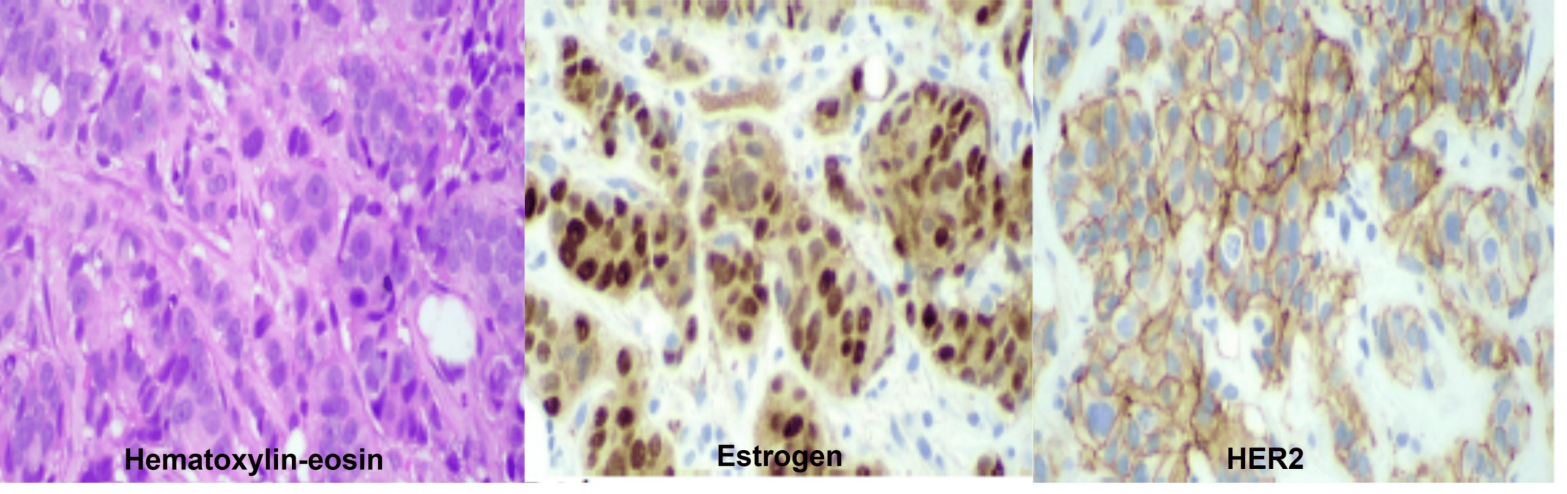
Immunohistochemical study from the left breast core needle biopsy Hematoxylin and eosin (H&E) staining showing an invasive epithelial neoplasm arranged in solid nests and cords infiltrating the fibrous stroma. The tumor cells display moderate nuclear pleomorphism, enlarged hyperchromatic nuclei, mitosis, and occasional prominent nucleoli, consistent with invasive ductal carcinoma. Estrogen receptor (ER) immunohistochemistry demonstrating strong and diffuse nuclear positivity in the majority of tumor cells, indicating a hormone receptor–positive phenotype. HER2 immunohistochemistry demonstrating a circumferential membranous staining pattern in the tumor cells, compatible with HER2 overexpression.

The initial immunohistochemical (IHC) evaluation revealed invasive carcinoma positive for estrogen and progesterone receptors, with equivocal HER2 expression (IHC score 2+). The interpretation of HER2 IHC Score 2+ indicates circumferential, weak-to-moderate membrane staining in >10% of tumor cells, or intense and complete membrane staining in 10% of tumor cells (Table 1 and Figure 2).

**Table 1:** Positive and negative controls used to attest to the fidelity of reactions through polymer-based detection. 6F11 - Specific monoclonal antibody to detect Estrogen Receptor 16 - Specific monoclonal antibody to detect the E-Cadherin protein SP3 - Specific monoclonal antibody to detect HER2 Oncoprotein K2 - Specific monoclonal antibody to detect Ki-67 (cell proliferation antigen) DAK-p63 - Specific monoclonal antibody to detect the p63 protein 26A11 - Specific monoclonal antibody to detect the GCDFP-15 protein (Gross Cystic Disease Fluid Protein-15).

Antibodies	Clone	Result	Observation
Estrogen receptor (ER)	6F11	Positive	+++, 90%
Progesterone receptor (PR)	16	Positive	+++, 50%
HER2 oncoprotein	SP3	Unsure	Escore 2+
Ki-67 (cell proliferation antigen)	K2	Positive	45%
p63 protein (squamous/transitional epithelia; myoepithelial cells)	DAK-p63	Negative	-
Calponin (smooth muscle and myoepithelial cells)	26A11	Negative	-

Therapeutic management and postoperative course

**Figure 3:**
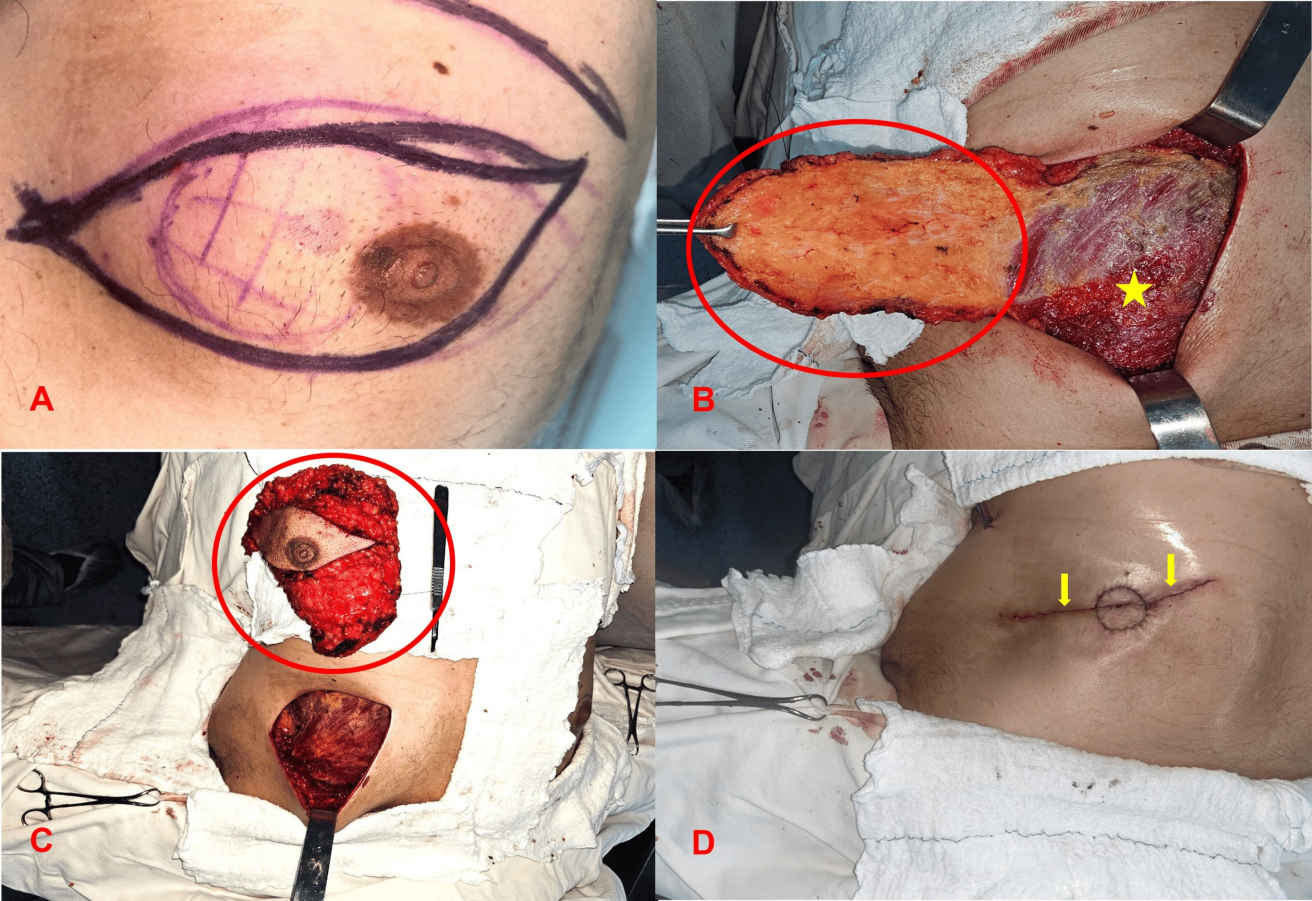
Left mastectomy (Sequence of surgical technique). A) Surgical marking B) Breast flap with tumor resection, revealing the pectoralis major muscle (*) C) Mastectomy specimen D) Continuous intradermal suture

The initial therapeutic approach included a left mastectomy (Figure 3) with the aim of maximizing the chances of cure and minimizing the risk of recurrence. This was considered the best option given the negative HER2 status and the absence of metastases on recent examinations.

On the first day of the immediate postoperative period, the patient experienced syncope upon standing to ambulate, bracing his right arm on a stretcher. One hour later, a significant hematoma was observed in the right breast, which had also undergone mastectomy (contralateral to the index surgery), necessitating an urgent return to the operating room to control bleeding caused by arterial rupture. The patient was discharged the following day with drains in both breasts.

On gross examination, the left mastectomy specimen weighed 306.0 g and measured 14.0 × 3.0 × 2.0 cm. The external surface was yellowish and lobulated, with sutures placed for margin orientation: two long stitches on the medial margin, two short stitches on the superior margin, and one long plus one short stitch on the lateral margin. The anterior surface demonstrated an ellipse of skin measuring 12.2 × 4.5 cm, with a nipple-areolar complex measuring 2.5 cm in diameter. On sectioning, a firm, whitish, well-circumscribed tumor with irregular borders was identified, measuring 2.0 × 1.9 × 1.7 cm and located 0.7 cm from the nipple and 1.5 cm from the closest (deep) margin. The remaining parenchyma was yellowish and lobulated, interspersed with whitish, elastic fibrous septa.

Additionally, three left sentinel lymph nodes with tan, smooth surfaces (largest 1.7 cm) and two left non-sentinel (parasentinel) lymph nodes with similar appearance (largest 0.7 cm) were submitted, all received within adipose tissue. 

**Figure 4:**
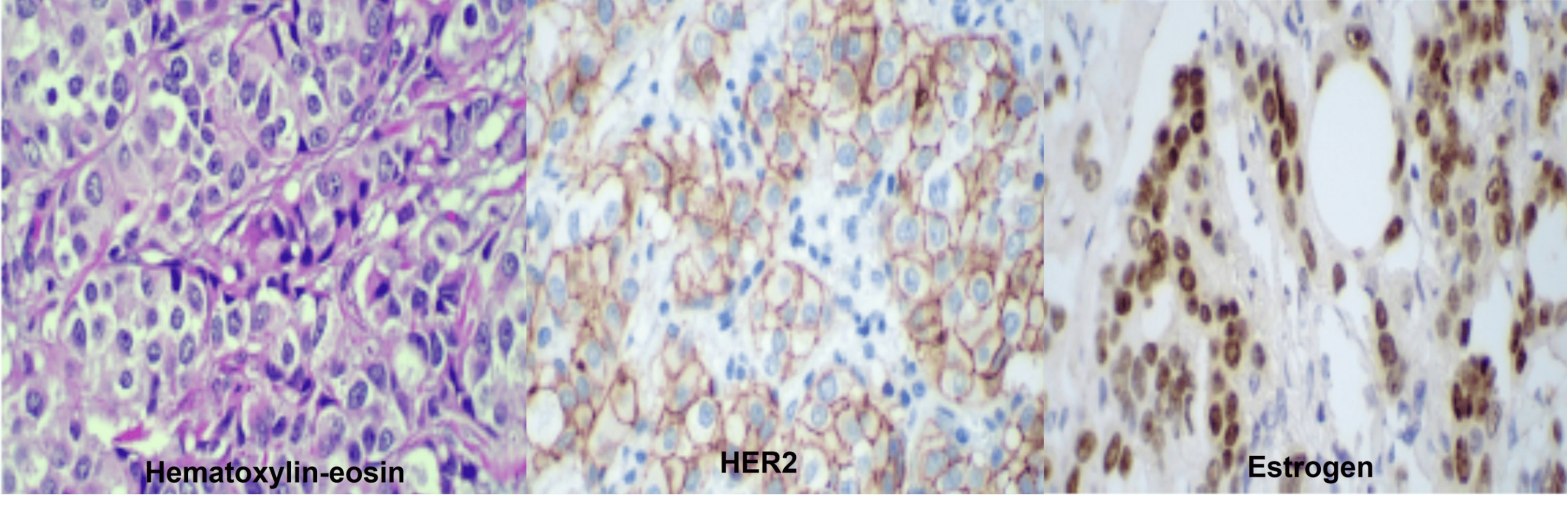
Immunohistochemical study after left mastectomy. Hematoxylin and eosin (H&E) staining revealed an invasive carcinoma composed of atypical epithelial cells arranged in solid nests. The tumor exhibits moderate nuclear pleomorphism, enlarged nuclei with coarse chromatin, and scattered nucleoli, features consistent with invasive ductal carcinoma. HER2 immunohistochemistry demonstrated complete membranous staining in the neoplastic cells, supporting HER2 overexpression. Estrogen receptor (ER) immunohistochemistry demonstrated diffuse and intense nuclear positivity in tumor cells.

Post-mastectomy histologic examination revealed invasive ductal carcinoma (no special type) measuring 2.0 cm in greatest dimension. The tumor was histologic grade III by the Nottingham system, with a nuclear grade of 3. Lymphovascular and perineural invasion were not identified. An associated ductal carcinoma in situ component was present, exhibiting a solid pattern with nuclear grade 3. Surgical margins were negative, with the closest margin (deep) measuring 1.5 cm from the tumor. Adjacent breast tissue demonstrated gynecomastia and stromal fibrosis. The nipple and adjacent skin showed no evidence of neoplastic involvement. No metastatic carcinoma was identified in the three sentinel lymph nodes or in the two left parasentinel lymph nodes. Pathologic staging according to the AJCC 8th edition was pT1c pN0(sn). The post‑mastectomy immunohistochemical study confirmed invasive carcinoma positive for estrogen and progesterone receptors and unsure (Score 2+) for HER2 (Table 2, Figure 4).

**Table 2:** Positive and negative controls used to attest to the fidelity of reactions through polymer‑based detection. 6F11 - Specific monoclonal antibody to detect Estrogen Receptor 16 - Specific monoclonal antibody to detect the E-Cadherin protein SP3 - Specific monoclonal antibody to detect HER2 Oncoprotein K2 - Specific monoclonal antibody to detect Ki-67 (cell proliferation antigen)

Antibodies	Clone	Result	Observation
Estrogen Receptor (ER)	6F11	Positive	+++, 90%
Progesterone Receptor (PR)	16	Positive	+++, 90%
HER2 Oncoprotein	SP3	Unsure	Escore 2+
Ki-67 (cell proliferation antigen)	K2	Positive	30%

Interpretation of immunohistochemical staining for the HER2 oncoprotein was performed according to the 2023 criteria established by the College of American Pathologists (CAP) [41]. A score of 0 (negative) was defined as the absence of staining or very faint, barely perceptible, and incomplete membrane staining in ≤10% of tumor cells. A score of 1+ (HER2-low) corresponded to incomplete, very faint or barely perceptible membrane staining in >10% of tumor cells. A score of 2+ (equivocal) was defined as circumferential, weak-to-moderate membrane staining in >10% of tumor cells, or intense and complete membrane staining in ≤10% of tumor cells. A score of 3+ (positive) required intense and complete circumferential membrane staining in >10% of tumor cells.

**Figure 5:**
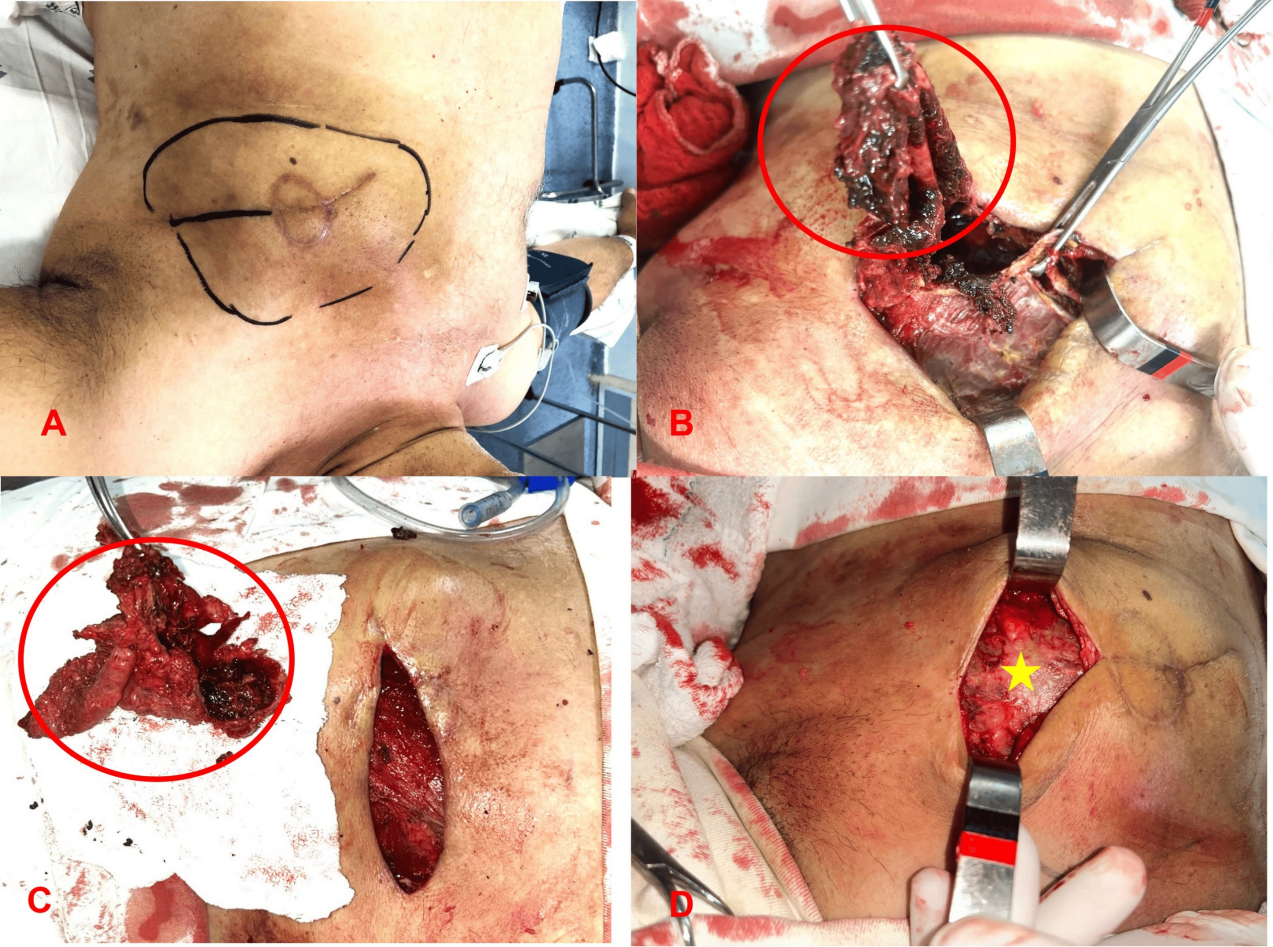
Resected fibrous capsule from the left breast seroma. A) Surgical marking; B) Fibrous capsule dissection; C) Resected fibrous capsule; D) Visualization of the pectoralis major muscle after resection of the fibrous capsule (*)

The drain was removed three weeks after surgery. Eight days later, the patient developed a seroma in the left breast, requiring 19 aspirations for evacuation, without control of fluid production. Sixty days after drain removal, a new intervention was necessary for resection of a fibrous capsule, which weighed 72 g and measured 11.3 × 8.5 × 3.5 cm (Figure 5). Microscopic examination revealed a cystic formation with walls composed of dense, organized, and well-vascularized fibrous tissue, with an internal surface lacking epithelial lining. The external surface of the capsule showed mature fibro‑adipose tissue without cytologic atypia, findings compatible with a well‑vascularized seroma wall and no residual neoplasia in the histologic sections examined.

Subsequently, the Oncotype DX Breast Recurrence Score assay was performed [42]. This test utilizes reverse transcription polymerase chain reaction (RT-PCR) to provide prognostic information and to estimate the magnitude of benefit from adjuvant chemotherapy. It plays a critical role in guiding treatment decisions for patients with early-stage, hormone receptor-positive (HR+) breast cancer, including both node-negative and selected node-positive cases, based on exploratory subgroup analyses from the TAILORx and NSABP B-20 trials evaluating absolute chemotherapy benefit for distant recurrence according to patient age and recurrence score. The Oncotype DX Breast Recurrence Score report for this node‑negative patient was 24. Given the patient’s age (>50 years), this result confers no meaningful benefit from chemotherapy (<1%). Consequently, endocrine therapy with tamoxifen citrate 20 mg daily for five years was selected.

## Discussion

The present case of MBC in a 64-year-old patient aligns with the characteristic epidemiologic profile of this rare neoplasm, which, despite accounting for a small percentage of breast cancer diagnoses, has shown a progressive increase in incidence over recent decades [1,2,4]. The patient’s age is consistent with the typical age at diagnosis, which most often occurs between 60 and 70 years [5,7-9].

The clinical presentation of MBC frequently involves a palpable, often painless breast mass predominantly located in the retroareolar region, which may be accompanied by skin retraction or nipple discharge [16,23]. In our patient, the presence of a palpable mass in the left breast with retraction of the nipple-areolar complex is consistent with these features described in the literature. Mammography revealed bilateral gynecomastia and a highly suspicious nodule (BI-RADS 5) measuring 4.3 cm, constituting a crucial diagnostic step. Although gynecomastia, which may signal hyperestrogenism, is recognized as a factor associated with MBC, the detection of a suspicious nodule and nipple retraction were the primary indicators warranting investigation for malignancy, underscoring that gynecomastia alone does not increase the risk of MBC [3,18,21,22].

Histopathologically, invasive ductal carcinoma (IDC), or no special type (NST), is the most common histologic subtype in MBC [1,2,24]. The patient’s diagnosis of invasive carcinoma of no special type (ductal), histologic grade III and nuclear grade 3, confirmed on both biopsy and post-mastectomy specimens, is consistent with the literature. High positivity for hormone receptors, specifically ER and PR (present in more than 90% of cases), is a distinguishing feature of MBC [1-3,24]. The patient showed strong ER (90%) and PR (50% initially, 90% post-mastectomy) positivity, consistent with a luminal molecular subtype, the most common in men. The low HER2 positivity and the rare incidence of triple‑negative tumors in MBC (typically <10% and <1%, respectively) [1-3,20] were also observed in this case, in which HER2, initially explicit (score 2+) by immunohistochemistry, was confirmed as non‑amplified by FISH, reinforcing this population characteristic.

Among the etiologic factors for MBC, hormonal imbalances, such as hyperestrogenism, frequently linked to Klinefelter syndrome, obesity, liver disease, and exposure to exogenous estrogens, and a positive family history are recognized as significant factors [9,20-22]. The patient’s bilateral gynecomastia may indicate hyperestrogenism, a relevant etiopathogenic factor for MBC [15,21,22]. Additionally, performing an NGS panel for hereditary breast cancer in this patient underscores the importance of genetic predisposition, given that mutations in genes such as BRCA2, BRCA1, PALB2, CHEK2, and ATM are well‑established risk factors. It is recommended that all men diagnosed with MBC be considered for genetic counseling and testing [2,14,17,21,24,34,36,37].

The prognosis of MBC is often less favorable than that of female breast cancer (FBC), mainly due to delayed diagnosis and presentation at more advanced disease stages [7,8,17]. However, the post‑mastectomy pathologic analysis in our patient revealed early‑stage disease (pT1c, pN0[sn]), which contrasts favorably with the general trend toward advanced stages at MBC diagnosis [8,23]. The absence of neoplastic involvement in the sentinel and non‑sentinel lymph nodes, along with negative surgical margins and the small tumor size (2.0 cm), are favorable prognostic factors for this patient [17,43].

Therapeutic management of MBC is predominantly guided by extrapolation from protocols established for female breast cancer (FBC), due to the rarity of the condition and the scarcity of randomized clinical trials specific to male patients [32-34,36]. Surgical intervention, with mastectomy as the most common technique, remains the cornerstone of local treatment [15,35,36]. Notably, male patients are significantly more likely to undergo mastectomy and less likely to be referred for radiotherapy after breast‑conserving surgery compared with female patients [6]. The left mastectomy performed in our patient followed the standard surgical approach.

The decision regarding adjuvant chemotherapy is often supported by genomic assays such as the Oncotype DX Breast Recurrence Score, which assesses recurrence risk and the expected benefit of chemotherapy, particularly in hormone‑dependent, node‑negative tumors [3,17,24]. In the present case, a recurrence score of 24 (for a node‑negative patient older than 50 years) indicated a chemotherapy benefit of less than 1%, supporting the decision to proceed with adjuvant endocrine therapy alone. Tamoxifen 20 mg daily for five years post-mastectomy remains the standard therapy for most hormone-dependent MBC tumors [15,35,36]. However, adherence to long‑term endocrine therapy, such as tamoxifen, remains a notable challenge, often due to associated adverse effects [38,39].

The complications observed in the immediate postoperative period, including syncope with hematoma formation in the contralateral breast and a persistent seroma in the operated breast that required multiple aspirations and resection of a fibrous capsule, underscore the complexity of surgical management and the need for vigilant monitoring. Seroma is a collection of serous fluid, resulting from the accumulation of blood plasma and/or lymph, and is a frequently observed complication in surgical interventions for breast cancer. Its incidence can reach up to 85% of cases, making it an almost inherent sequela of mastectomy. The magnitude of seroma is quite variable, requiring interventional management in some situations and allowing a conservative approach in others [44]. According to Subramanian et al, postoperative drain removal is indicated by breast surgeons when the seroma output in the preceding 24 hours is between 30 and 50 mL [45]. Prolonged drainage, associated with continuous seroma accumulation, leads to a longer hospital stay and increased infection risk, with the shortest period for drain removal being 4 to 5 days. These events highlight the unpredictability of certain surgical sequelae and the importance of rigorous, multidisciplinary follow‑up.

This complex clinical scenario and the challenges encountered during follow‑up reinforce the pressing need to increase awareness both among the general population and healthcare professionals, as well as to ensure ongoing investment in research dedicated to MBC. Such efforts are crucial to enhance early diagnosis, refine personalized therapeutic strategies, and thereby optimize the prognosis and quality of life of male patients affected by this rare disease [33,34,36].

## Conclusions

This case reinforces the complex and often underestimated nature of MBC. The swift diagnostic investigation, which culminated in early-stage disease, was a crucial determinant for this patient's favorable prognosis, contrasting sharply with the general tendency for delayed diagnoses and less promising outcomes often observed in MBC cases.

These findings underscore the critical importance of early diagnosis, which also informs prevention strategies through the recognition and monitoring of risk factors. Therefore, it is imperative to intensify public awareness, enhance healthcare professional training, and promote sustained research and specific care guidelines for men. These concerted efforts are essential not only to optimize clinical management but also to significantly improve the prognosis and overall experience of men affected by this rare disease.
